# Weaning age impacts intestinal stabilization of jejunal intraepithelial T lymphocytes and mucosal microbiota in pigs

**DOI:** 10.1186/s12917-025-04850-5

**Published:** 2025-07-19

**Authors:** Jayne E. Wiarda, Hannah R. Watkins, Melissa S. Monson, Christopher L. Anderson, Crystal L. Loving

**Affiliations:** 1 Food Safety and Enteric Pathogens Research Unit, National Animal Disease Center, Agricultural Research Service, United States Department of Agriculture, Ames, IA USA; 2 Oak Ridge Institute for Science and Education, Agricultural Research Service Participation Program, Oak Ridge, TN USA; 3 Department of Veterinary Microbiology and Preventative Medicine, College of Veterinary Medicine, Iowa State University, Ames, IA USA; 4 Present Address: Virus and Prion Research Unit, National Animal Disease Center, Agricultural Research Service, United States Department of Agriculture, Ames, IA USA

**Keywords:** Intraepithelial T lymphocytes, Jejunum, Mucosal microbiota, Weaning, Intestinal immunity

## Abstract

Weaning in conventional pig production is a stressful event that involves abrupt dietary and environmental changes, and the post-weaning period is associated with increased incidence of disease and antibiotic use. As a result, there is a growing demand for non-antibiotic practices to enhance health during this phase of production. Current production systems wean piglets at a relatively young age, and it is unclear if age at weaning impacts shifts in intestinal immune populations, particularly intraepithelial T lymphocyte (T-IEL) populations, or bacterial communities, in a comparable timeframe and magnitude. T-IELs reside in the intestinal epithelium and play a role in intestinal integrity and defense. While later weaning may be an approach to minimize the negative impacts of weaning, the impact of age at weaning on T-IEL abundances and function is not clearly understood. Our results indicate pigs weaned at 18–21 days of age (doa; standard weaned, SW), which is consistent with production practices in the United States, had delayed shifts in T-IEL populations when compared to pigs weaned at 25–28 doa (late weaned, LW), which is consistent with practices in the European Union. Specifically, the abundance of induced T-IELs (CD8αβ^+^ αβ T cells) in the jejunum increased between 0 and 3 days post-weaning (dpw) for the LW group and stabilized, but shifts were delayed until between 3 and 7dpw in the SW group. Concomitant decreases in the abundance of natural T-IEL (γδ T cells) were detected. The structure of jejunal mucosal bacterial communities were comparable between SW and LW pigs at 0dpw, but the SW group had more pronounced shifts from 3 to 7dpw and 7dpw to 14dpw compared to minimal shift in LW group from 7 to 14dpw. Body weight between groups was comparable when adjusted for age. Overall, the bacterial communities of the jejunal mucosa were more stable after weaning in LW than SW pigs, and jejunal T-IEL abundances stabilized more rapidly in the LW group. As T-IELs play a key role in intestinal homeostasis and barrier integrity, the early differences in population abundance may be indicative of functional differences as well.

**Supplementary Information:**

The online version contains supplementary material available at 10.1186/s12917-025-04850-5.

## Introduction

Intraepithelial T lymphocytes (T-IELs) are immune cells residing within the intestinal epithelium that serve as first-line defenders against enteric microbes while simultaneously promoting intestinal barrier integrity and tolerance to commensal microbes [1–5]. As some of the earliest immune populations present in the intestinal tract, T-IELs have important roles in shaping the early and long-term intestinal immune environment [6]. The two characterized subsets of T-IELs are referred to as natural T-IELs and induced T-IELs (reviewed in [7]). Naturally-occurring resident T-IEL populations in the intestine include γδ T cells and CD8αα^+^ αβ T cells, while induced T-IELs include CD8αβ^+^ αβ and CD4 αβ T cells that are activated by antigen in the periphery and then home to the intestinal tract [8, 9]. Pig T-IEL populations are primarily comprised of γδ T cells (natural T-IELs) and CD8αβ^+^ αβ T cells (induced T-IELs) at weaning, with differences across intestinal locations and expected shifts noted after weaning [10, 11]. In mice, induced T-IELs accumulate in response to dietary and microbial antigens associated with weaning, which is expected given conventional MHC-mediated activation (reviewed in [7]). Natural T-IELs are present at birth and decrease with age, while induced T-IELs increase with antigen exposure [12]. Dysregulation of the balance between immune activation and regulation by T-IELs can have detrimental effects attributed to immune activation by microbe- and/or diet-derived antigen exposure (reviewed in [6, 7]).

Microbiota, diet, and stress levels can undergo dynamic shifts in early life, leading to long-term impacts on the intestinal immune system and potentially cause intestinal immunopathology [reviewed in [13–18]]. In absence or reduction of microbial members colonizing the intestinal tract (i.e. germ-free or antibiotic-treated mice), T-IEL numbers are reduced, and diminished cell motility, cytokine production, and antimicrobial protein production is observed [1, 4, 5, 19], demonstrating T-IEL prevalence and function is also shaped by the microbiota. In absence or reduction of dietary antigen (i.e. animals fed minimal ingredient or antigen-free diets), diminished T-IEL antimicrobial protein production and cytotoxicity is observed in association with epithelial damage and increased bacterial burden [20–23]. Dietary change affects gut microbial composition, which may have lasting influences on health, productivity, and disease resistance [24, 25]. Collectively, microbiota, diet, stress, and immune components such as T-IELs are all members of an interlinked network that contributes to intestinal and overall health outcomes, and while well studied in murine models, such interactions remain understudied in pigs.

Current practices in the pig industry involve weaning animals at a relatively young age, resulting in shifts to the microbiota, increased stress, abrupt dietary change, and compromised intestinal barrier integrity [15, 18, 26–30] – all factors known to affect T-IEL prevalence and behavior in the intestine. In the swine industry, a major goal is to limit post-weaning intestinal distress and disease, which can be achieved through various intervention strategies designed to stabilize microbial and immune communities, thus minimizing intestinal perturbations [31, 32]. However, the functional dynamics of T-IELs following pig weaning and resultant influences on short- and long-term intestinal and overall health are only beginning to be understood. T-IELs in the pig intestine undergo dramatic shifts in abundance, phenotype, proliferation, activation, and metabolism immediately following weaning [reviewed by [33]]; however, the effects of intervention strategies on T-IEL abundance and function, and interlinkage with factors such as the bacterial microbiota, in the post-weaning period are poorly defined.

Pigs are usually weaned no earlier than 28 doa in most European countries [34], yet weaning in the United States often occurs earlier, at ~ 18–21 doa [35]. Compared to pigs weaned at 18–21 doa, pigs weaned at 28–30 doa have enhanced barrier integrity [36, 37], increased microbial diversity [38], reduced severity of microbial disruption [39], reduced destructive behavior [40], decreased levels of stress hormone [40, 41], decreased likelihood of requiring antibiotic treatment later in life [42], and less intestinal inflammation [36, 37] in the immediate post-weaning period. Thus, later weaning may be an intervention strategy used to minimize negative weaning impacts and an alternative to in-feed antibiotics [reviewed in [43–45]. However, the impact of weaning age on T-IEL abundances and function is not well established, particularly in relation to shifts in the intestinal microbial communities. Thus, we assessed the compositions of jejunal T-IEL communities in the intestine of pigs weaned at 18–21 or 25-28doa at various timepoints with comparisons matched for pig age or dpw. Because of microbiota-T-IEL interlinkage and the important implications of weaning age on pig weight, we also analyzed mucosal bacterial communities in the pig jejunum and pig body weights at matched timepoints.

## Materials and Methods

### Animals and sample collection

Conventional, mixed white pigs were farrowed from sows in a synchronized manner in an indoor facility at the ARS-Meat Animal Research Center in Clay Center, Nebraska. Litters were timed to wean at ages indicated below. Piglets did not receive any creep feed while with the sow. Weights were recorded one day prior to weaning (denoted as 0dpw* in the paper) and the next morning weaned and transported to the ARS-National Animal Disease Center (NADC) in Ames, Iowa. Animals in the standard-weaned (SW) group were derived from seven sows (*n* = 48 piglets), and weaned between 18–21 doa; animals in the late-weaned (LW) group were derived from five sows (*n* = 40 piglets) and weaned between 25-28doa. Upon arrival to the NADC, pigs for each respective group (LW or SW) were each split across two BSL2 isolation rooms (no penning), each room measuring 13.4m^2^/144 ft^2^. Each room was equipped with four nipple waterers and two feeders with rations added twice per day. Pigs were fed a full ration (based on pig weight) of Ultracare® Starter 1 (Purina® Animal Nutrition, Arden Hills, MN) for approximately 14 days and transitioned to 2075 NexGen® Advanced 14 Complete (Kent Nutrition Group, Mucatine, IA) finisher diet for the remainder of the study. No antibiotics were included in the diet and animals remained healthy throughout the trial (no parenteral antibiotics were warranted or administered). Within the SW and LW treatment groups, variables of gender and starting weight were randomly distributed across animal rooms and necropsy dates. Animal meta data are available from repositories listed in the Data Availability section.

**Fig. 1:**
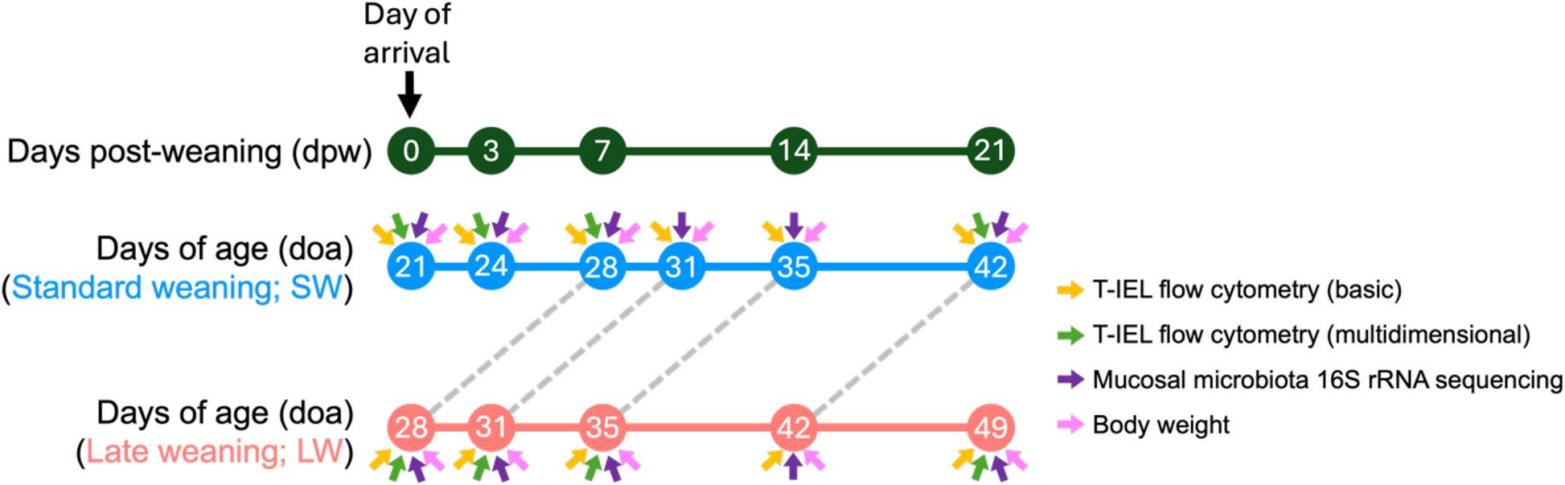
Experimental overview. Experimental timeline indicating days post-weaning (dpw; dark green timeline) and days of age (doa; blue and red timelines) when samples were collected and used for study comparisons. A light grey dotted line connects age-matched samples collected from standard weaned (SW; blue) and late weaned (LW; red) timelines. Above the doa timeline for SW pigs (blue timeline) and below the doa timeline for LW pigs (red timeline), arrows are shown indicating which samples were collected for each timepoint, including collection of jejunal T-IELs for basic flow cytometry compositional analysis (gold arrows), collection of jejunal T-IELs for multidimensional flow cytometry compositional analysis (green arrows), collection of jejunal mucosal microbiota samples for compositional analysis (purple arrows), and recording of animal body weights (pink arrows). Measurements were collected from each of the animals necropsied from each treatment group at the specified timepoints (*n* = 8 per treatment group per timepoint noted). Abbreviations: rRNA (ribosomal RNA); T-IEL (intraepithelial T lymphocyte)

Pigs were humanely euthanized using intravenous administration of sodium barbiturate to effect (cessation of breathing and heartbeat). Tissues were collected at various timepoints matched to dpw or doa between SW and LW pigs, as shown in Fig. 1. A total of 8 pigs per group (SW and LW) were euthanized at each time point, with half of the animals in a group coming from each one of the two isolation rooms to minimize room effect. Jejunum was located by evaluating small intestinal tissue 9–12 feet proximal to the ileocecal junction and confirming a lack of discrete Peyer’s patches (which is indicative of ileum). Two ~ 3-inch sections of jejunum were collected for epithelial cell isolation (cell phenotyping by flow cytometry) and mucosa collection (bacterial 16S rRNA gene sequencing).

### Animal weight data recording and analysis

Animal weights were recorded one day prior to weaning (before transport; 0dpw*/28doa*) and immediately prior to euthanasia (timepoints shown in Fig. 1). Statistical analyses of weight data were performed using Prism v9.4.1 (GraphPad Software, San Diego, CA, USA) as described below.

A non-parametric Kruskal–Wallis test was used to compare preweaning (before transport) weights amongst pigs necropsied at different timepoints within either the SW or LW groups.

Weights were further compared between SW and LW pigs at dpw- or doa-matched timepoints using non-parametric Kruskal–Wallis testing and multiple comparison Dunn’s testing for selected pairwise comparisons. As only pre-determined pairwise comparisons were performed, *p*-values were not corrected for multiple testing. A *p*-value < 0.05 was considered significant, with *p*-values < 0.1 noted also in the Figure. A simple linear regression was also fit to necropsy weight and ADG data based on exact doa in order to test for significantly different slopes of data (indicating significantly different rates of increase/decrease over time) and significantly different y-intercepts (indicating significantly different starting values). A *p*-value < 0.05 was considered significant.

### Cell isolations for flow cytometry

All reagents were equilibrated to room temperature before use. In the barn, jejunal tissue for cell isolations was placed into 30 mL Hank’s balanced salt solution (HBSS) containing 2 mM ethylenediaminetetraacetic acid (EDTA; Invitrogen AM9260G; Waltham, MA, USA), 2 mM L-glutamine (Gibco 25–030; Waltham, MA, USA), and 0.5% bovine serum albumin (BSA; Sigma-Aldrich A9418; St. Louis, MO, USA). In the lab, ~ 2 g of jejunal tissue was dissected and used for cell isolations. Weighed tissue was placed into 30 mL HBSS containing 5 mM dithiothretol (Invitrogen 15,508) and 2% fetal calf serum (FCS; heat-inactivated in-house; Gibco A38401) and placed in a shaking incubator Thermo Scientific MaxQ 4000 (Thermo Fisher, Waltham, MA, USA) at 200 rpm at 37 °C for 20 min. Tissues were transferred into another 30 mL HBSS containing 5 mM EDTA and 2% FCS and placed on a shaking incubator at 37 °C for 20 min. Transfer and incubation with fresh solutions of 5 mM EDTA and 2% FCS in HBSS were repeated for a total of three incubations. Tissue was discarded following the final incubation. Tubes from the three incubations were centrifuged 10 min 300 g at room temperature to pellet liberated epithelial cells. Cell pellets were resuspended in HBSS containing 2 mM L-glutamine and 2% FCS, filtered through a 100-micron nylon filter, and cell viability assessed with a Muse Count & Viability Assay Kit (Luminex MCH100102; Austin, TX, USA) on a Muse Cell Analyzer (Luminex 0500–3115). Cells were maintained at room temperature and immediately used for flow cytometry staining.

### Flow cytometry staining and data acquisition

Staining for extracellular surface markers was performed as previously described [10]. All cells were incubated with viability dye (Fixable Viability Dye-eFluor780; Thermo Fisher Scientific 65–0865-14), anti-CD2 (clone PG168A; Washington State University [WSU] 65–0865-14; Pullman, WA, USA) detected with anti-mouse IgG2a-BV711 (clone R19-15; BD Biosciences [BD] 744,533; Franklin Lakes, NJ, USA), anti-CD3ε-PE-Cy7 (clone BB23-8E6-8C8; BD 561477), anti-CD4-PerCP-Cy5.5 (clone 74–12-4; BD 561474), anti-CD8β-PE (clone PPT23; BioRad MCA5954PE; Hercules, CA, USA), anti-CD21-BV650 (clone B-ly4; BD 740569), and anti-γδTCR-mFluor510 (clone PGBL22A; WSU PG2032; custom-conjugation to mFluor510 performed by Caprico Biotechnologies; Norcross, GA, USA).

An extended phenotype panel was performed for a subset of timepoints (0, 3, 7, and 21dpw) and included labeling for anti-CD8α-AF647 (clone 76–2–11; BD 561475), anti-CD16 (clone G7; BioRad MCA1971GA) detected with anti-mouse IgG1-BUV395 (clone A85-1; BD 740234), anti-CD27-FITC (clone B30C7; BioRad MCA5973F), anti-CD45RC (clone MIL5; BioRad MCA1750) detected with anti-mouse IgM-BV421 (clone Il/41; BD 743323), and anti-MHC-II (MHC II isotype SLA-DR; clone 2E9/13; BioRad MCA2314GA) detected with anti-mouse IgG2b-BUV496 (clone R12-3; BD 750517). Single stain, fluorescence-minus-one controls, and a cryopreserved sample of ileal epithelial-enriched cells from a conventional six-week-old pig was also included for labeling at each timepoint as a batch control.

Data was acquired on a FACSymphony A5 flow cytometer (BD Biosciences) using instrument settings within recommended ranges and setting and appropriate hardware voltage and compensation set using UltraCompe eBeads™ Plus Compensation beads (ThermoFisher 01–333-41), single stain, and fluorescence-minus-one controls as previously described [10].

### Basic cell phenotyping

**Fig. 2:**
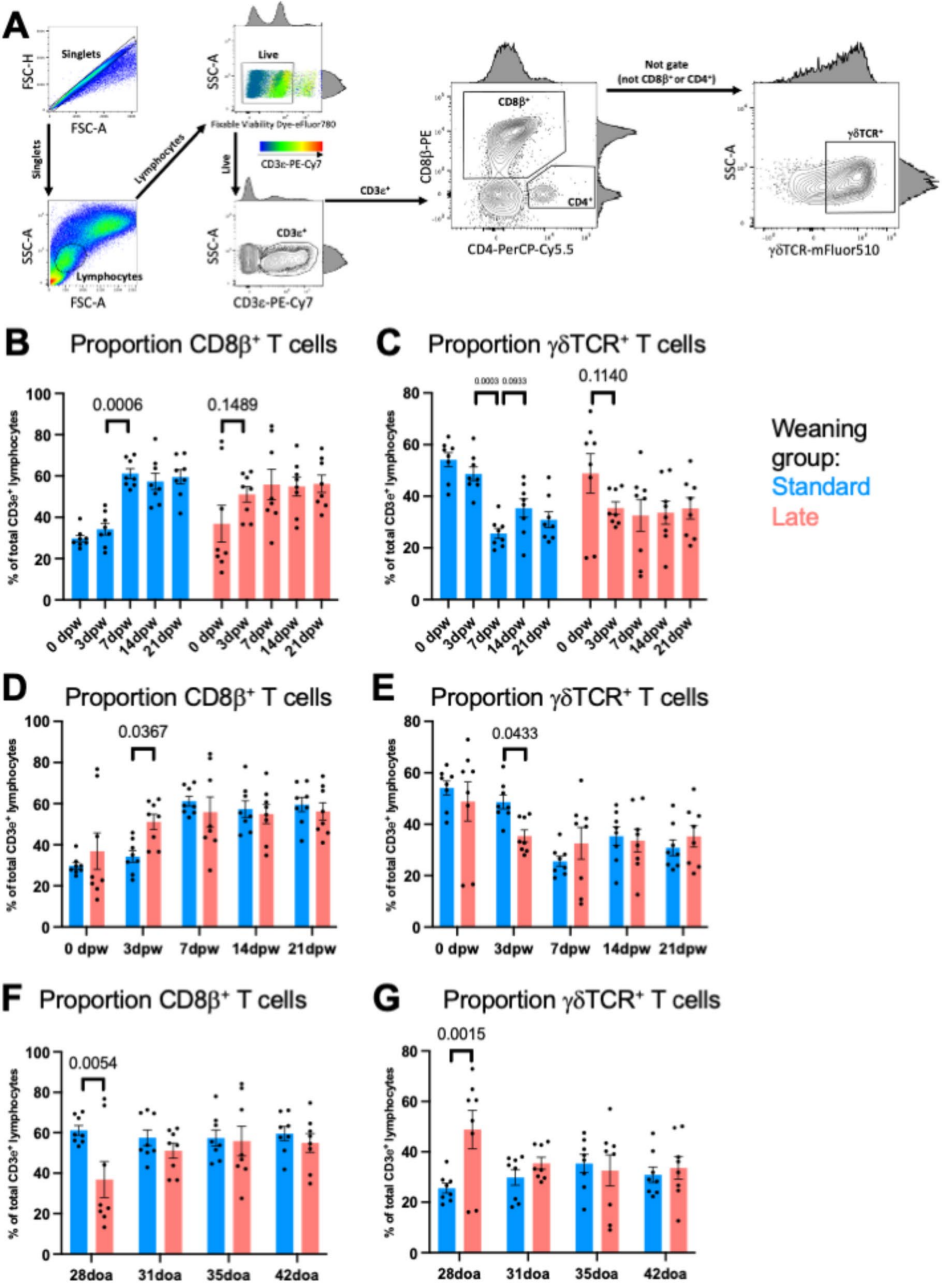
Alterations in gd and CD8b ab T cell proportions of the jejunal epithelium from standard- and late-weaned pigs. **A** Gating strategy used to identify T cell subsets from samples of jejunal epithelial-enriched cell fractions analyzed via flow cytometry. **B** Comparison of the proportion of CD8β^+^ T cells in sequential dpw timepoints of standard- (blue) or late- (red) weaned pigs. **C** Comparison of the proportion of γδTCR^+^ T cells in sequential dpw timepoints of standard- (blue) or late- (red) weaned pigs. **D** Comparison of the proportion of CD8β^+^ T cells in dpw-matched timepoints of standard- (blue) versus late- (red) weaned pigs. **E** Comparison of the proportion of γδTCR^+^ T cells in dpw-matched timepoints of standard- (blue) versus late- (red) weaned pigs. **F** Comparison of the proportion of CD8β^+^ T cells in doa-matched timepoints of standard- (blue) versus late- (red) weaned pigs. **G** Comparison of the proportion of γδTCR^+^ T cells in doa-matched timepoints of standard- (blue) versus late- (red) weaned pigs. Statistical comparisons in B-E were performed with a Kruskal–Wallis test then Dunn’s test for selected pairwise comparisons. All *p*-values < 0.15 are shown. Pairwise comparisons were made between standard- versus late-weaned pigs at matched dpw timepoints in B, D,between sequential dpw timepoints within standard- or late-weaned pig groups in C, E, and between standard- versus late-weaned pigs at matched doa timepoints in F, G. Abbreviations: dpw (days post-weaning); doa (days of age); FSC-A (forward scatter-area); FSC-H (forward scatter-height); SSC-A (side scatter-area)

FlowJo v10.8.1 (BD Biosciences) was used to identify viable, CD3ε + lymphocytes as T-IELs, similar to previous work [10, 11] and further gate into T-IEL subsets based on expression of CD4, CD8β, and γδTCR (Fig. 2A). Percentages of different T-IEL subsets within total T-IELs were statistically compared between dpw- or doa-matched pigs of SW and LW treatment groups, as well as between sequential dpw timepoints within SW or LW groups using Prism v9.4.1. The non-parametric Kruskal–Wallis test was used to compare main effects followed by multiple comparisons Dunn’s testing for selected pairwise comparisons. As only pre-determined pairwise comparisons were performed, *p*-values were not corrected for multiple testing. An alpha level for significance was not set, but only *p* < 0.15 are presented in results.

### Flow cytometry high-dimensional analysis

For samples labeled with extended cell phenotype markers (described in above methods), T-IEL populations (viable, CD3ε + lymphocytes) were identified using manual gating in FlowJo. Gated T-IELs were then exported from FlowJo as separate.fcs files containing compensated fluorescence intensity values for cell markers and uploaded into R v4.2.2 for further analysis. Files were merged to create a data table where each row represented one cell, and parameters of compensated fluorescence intensity values for cell markers and sample meta data were listed in columns.

Fluorescence intensity values were transformed based on the Spectre workflow (Spectre v1.0.0; [46]) using optimized arcsin transformations (cofactors 100, 250, 500, 750, and 1000 tested). Batch correction of transformed fluorescence intensity values was performed using the coarse alignment protocol of the CytofBatchAdjust algorithm based on a workflow outlined by Spectre and specifying the cryo-thawed ileum sample included in all staining timepoints as the batch control. Batch-corrected data tested with different arcsin transformations was plotted to identify an optimal transformation for each batch-corrected marker that was used for further analyses. Batch control samples were removed from further analyses.

Using the Spectre package, clustering with FlowSOM [47] was performed to identify and remove non-T cell events (low fluorescence intensity values for all cell markers) as previously described [11]. Filtered data were further reduced to include equal numbers of cells randomly selected from each sample (*n* = 1,147 cells per sample; 73,408 cells total across 64 samples).

Filtered data were converted to a Seurat object (Seurat v4.3.0.1; [48]), and dimensionality reduction was performed to create a uniform manifold approximation projection (UMAP) plot based on principal components calculated for the data. Data were converted into a milo object and analyzed with miloR v1.4.0 [49] to perform differential abundance testing with a negative binomial generalized linear model with spatially-corrected false discovery rates (FDRs). Cells were divided into cell neighborhoods using the graph-based reduction method, and differential abundance tests were completed between doa- or dpw-matched treatment groups of SW versus LW pigs or between consecutive timepoints within SW or LW treatment groups. Confounding variables of gender, litter, preweaning weight, and variation from targeted age were included in the statistical testing design when possible and not nested within another variable. Weaning age group, gender, and variation from targeted doa were included in the statistical model for differential abundance testing between doa- and dpw-matched SW versus LW groups. Weaning age group, gender, litter, preweaning weight, and variation from targeted doa were included in the statistical model for testing between consecutive timepoints within SW or LW treatment groups. FDRs < 0.05 were considered significant.

### Mucosal bacterial DNA extraction and 16S rRNA gene sequencing

Sections of jejunum were cut longitudinally, opened, and gently rinsed in PBS (pH 7.2) to remove luminal contents. A mucosal sample was collected by rotating the head of a sterile polyester swab (Puritan™ 25–806 2PD; Guilford, ME, USA) across the rinsed jejunal section for approximately 10 s. The swabs were placed into 2 mL of chilled PBS in a 15 mL falcon tube and stored on ice to transport to the lab. In the lab, swabs were vortexed vigorously for 30 s and stored at −80 °C across multiple aliquots. A 0.25 mL aliquot of the resulting PBS solution was used for DNA extraction with the ZymoBIOMICS 96 MagBead DNA Kit (Irvine, CA, USA). 16S rRNA gene amplicons of the V4 region were generated from jejunal mucosal DNA in accordance with the protocol described by Kozich et al. (30 cycle PCR) [50]. In addition to mucosal samples, blank DNA extractions and no template PCR reactions were prepared for sequencing. The V4 16S rRNA gene amplicons were sequenced on an Illumina Miseq (La Jolla, CA, USA) using the V2 reagent kit (2 × 250 paired-end reads).

### Mucosal bacterial community analysis

The V4 16S rRNA gene amplicon sequences were filtered, trimmed, and delineated into amplicon sequence variants (ASVs) using DADA2 v1.26 (default parameters) [51]. Information was pooled across samples to increase the sensitivity of amplicon variant discovery during sample inference portion of the DADA2 workflow. Taxonomy was assigned to ASVs using the IDTAXA algorithm of the DECIPHER v2.18 R package with the SILVA database (r138) [52]. ASVs not assigned to Domain Bacteria were removed from the analysis.

The Aitchison distance between bacterial communities was calculated using the auto-rpca function of the Deicode QIIME2 plugin [53, 54]. ASV counts are transformed based on a robust-centered log ratio as part of the Robust Aitchison PCA analysis. The resulting distance matrix was used in a PERMANOVA analysis within the vegan package (version 2.6–4, https://CRAN.R-project.org/package=vegan) to assess statistical differences between microbiomes based on weaning age group (standard or late), days of age, sex, and farrowing pen. Subsequent pairwise differences were tested using the pairwiseAdonis function. Multiple pairwise comparisons were corrected for using the false discovery rate (FDR) approach.

### Data availability

Scripts used for computational analyses of flow cytometry data are available at https://github.com/SwiVi/FlowCytometry_HighDimAnalysis_TIELs_WeaningAge. Scripts used for computational analyses of 16S rRNA sequencing data are available at https://github.com/USDA-FSEPRU/fs24_16S_jejunum. All raw sequence data has been deposited in the SRA and is available under BioProject PRJNA1199683.

## Results

### Impact of weaning age on intraepithelial T lymphocyte communities

To determine how later weaning affects T-IEL communities in the small intestine, epithelial-enriched cell fractions were isolated from the jejunum of pigs weaned at 18–21 doa (SW pigs) or 25-28doa (LW pigs) with doa and dpw-matched timepoints (*n* = 8 pigs/weaning age/timepoint) (Fig. 1). To identify T-IEL subsets, isolated epithelial-enriched jejunal cells were processed for cell labeling and assessment of cell populations by flow cytometry (Fig. 2A).

Similar to previous studies [10, 11], CD8αβ + αβ T-IELs (CD8β^+^) and γδ T-IELs (γδTCR^+^) comprised the majority of T-IELs (CD3ε^+^ lymphocytes) with CD8β and γδ T-IEL proportions increasing and decreasing with age/post-weaning time, respectively (Fig. 2B-C). Although the proportion of CD8β T-IELs increased over time in both SW and LW pigs, the largest increase (determined by smallest *p*-value) occurred earlier in LW pigs, between 0 to 3dpw (*p* = 0.1489) compared to a later increase between 3 to 7dpw for SW pigs (*p* = 0.0006) (Fig. 2B). A commensurate decrease in γδ T-IELs was similarly observed between 0 and 3dpw for LW pigs (*p* = 0.1140) compared to between 3 and 7dpw (*p* = 0.0003) for SW pigs (Fig. 2C). Greater proportions of CD8β T-IELs (*p* = 0.0367; Fig. 2D) and lower proportions of γδ T-IELs (*p* = 0.0433; Fig. 2E) were detected in LW compared to SW pigs at 3dpw, while at 28doa, LW pigs (0dpw) had lower proportions of CD8β T-IELs (*p* = 0.0054; Fig. 2F) and higher proportions of γδ T-IELs (*p* = 0.0015; Fig. 2G) compared to SW pigs (7dpw).

**Fig. 3:**
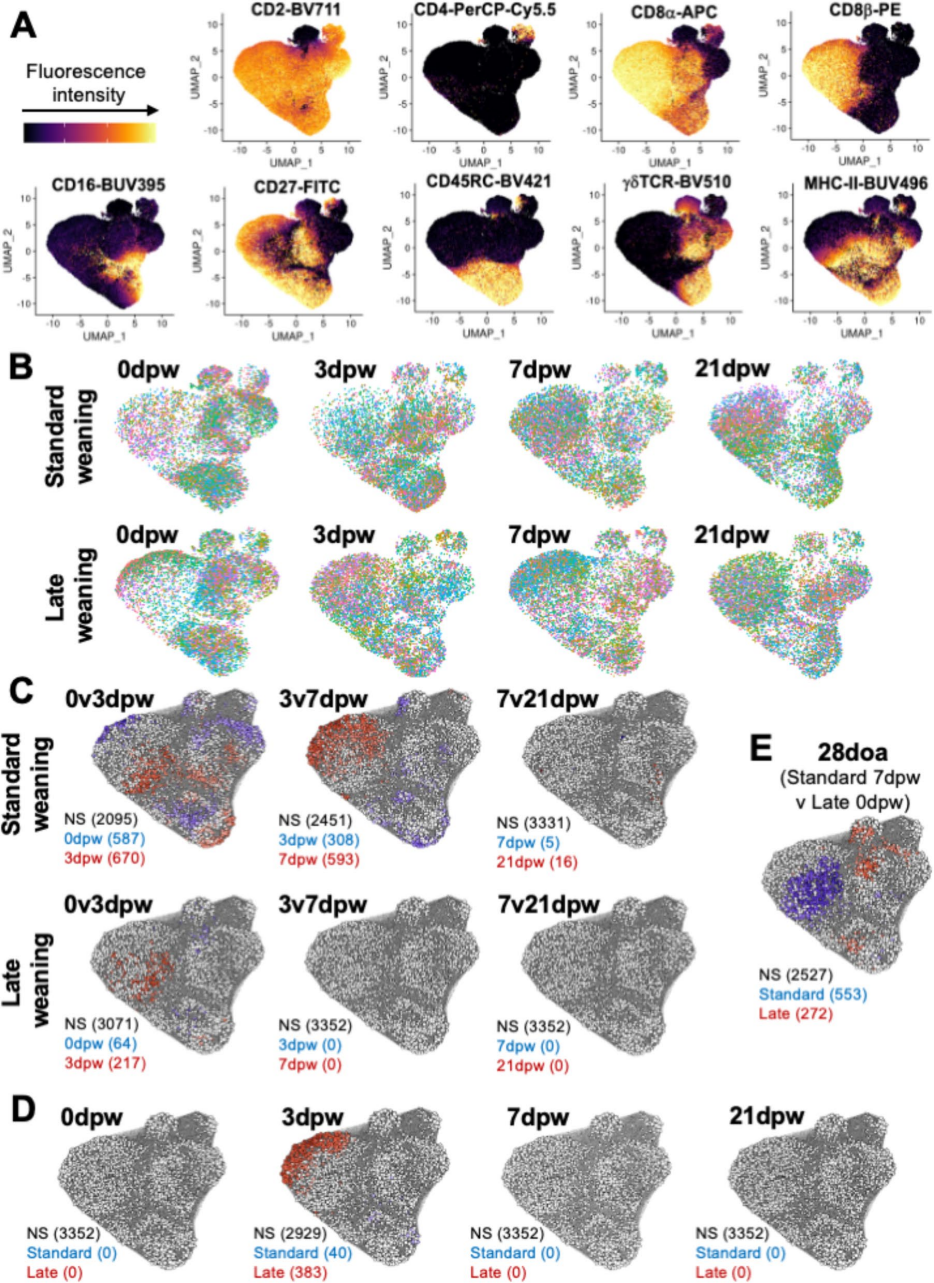
Differential abundance of T cell populations in the jejunal epithelium from standard- and late-weaned pigs. **A** UMAP plots displaying relative fluorescence intensity values for cell surface markers assessed via flow cytometry. Each dot represents one cell. Dot color corresponds to fluorescence intensity value of the cell surface marker represented within each respective panel. **B** UMAP plots displaying the distribution of cells derived from each treatment group. Each dot represents one cell. Dots are displayed in eight colors for each plot, corresponding to eight animals sampled in each treatment group. The same color seen across different plots does not correspond to the same animal. **C** Results of differential abundance testing in sequential dpw timepoints of standard- (top row) or late- (bottom row) weaned pigs. Each circle represents a cell neighborhood overlayed onto UMAP coordinates. Cell neighborhoods in blue have significantly greater abundance of cells in the earlier dpw timepoint. Cell neighborhoods in red have significantly greater abundance of cells in the later dpw timepoint. **D** Results of differential abundance testing in dpw-matched timepoints of standard- versus late- weaned pigs. Each circle represents a cell neighborhood overlayed onto UMAP coordinates. Cell neighborhoods in blue have significantly greater abundance of cells in standard-weaned pigs. Cell neighborhoods in red have significantly greater abundance of cells in late-weaned pigs. **E** Results of differential abundance testing in standard- versus late- weaned pigs at age-matched 28doa. Each circle represents a cell neighborhood overlayed onto UMAP coordinates. Cell neighborhoods in blue have significantly greater abundance of cells in standard-weaned pigs. Cell neighborhoods in red have significantly greater abundance of cells in late-weaned pigs. Statistical analysis for differential abundances was performed using a negative binomial generalized linear model test with spatially-corrected FDRs. A corrected FDR < 0.05 was considered significant. Abbreviations: doa (days of age); dpw (days post-weaning); FDR (false discovery rate); NS (not significant); UMAP (uniform manifold approximation projection)

To better understand more specific features of T-IELs that are affected by weaning age in the pig intestine, isolated jejunal epithelial-enriched cells collected at 0dpw, 3dpw, 7dpw, and 21dpw from both SW and LW pigs were labeled and analyzed via flow cytometry using a larger panel of antibodies reactive to cell surface markers, some indicative of function. To take a less biased approach to identifying affected T-IEL subsets, an annotation-independent method was utilized to analyze differential abundance of T-IELs in dpw- or doa-matched SW versus LW pigs as well as between sequential timepoints within each weaning age group. Cells were arranged in multidimensional space according to their fluorescence intensity profiles for nine cell surface markers (CD2, CD3ε, CD4, CD8α, CD8β, CD16, CD27, CD45RC, γδTCR, and MHC II) (Fig. 3A), comprising a dataset of 73,408 cells with equal numbers distributed across each pig/treatment group (Fig. 3B). Cells with similar profiles in multidimensional space were grouped together into cell neighborhoods (Supplementary Fig. 1), and statistical testing of cell abundance between different treatment group comparisons was conducted within each cell neighborhood. Differential abundance testing revealed significant differences (FDR corrected *p* < 0.05) in T-IEL community compositions within cell neighborhoods of LW pigs occurred only between 0 to 3dpw (281 neighborhoods), while significant shifts in SW pigs were prolonged, occurring not only between 0 to 3dpw (1257 neighborhoods) but also between 3 to 7dpw (901 neighborhoods) and to a smaller extent between 7 to 21dpw (21 neighborhoods) (Fig. 3C). Similar to results of Fig. 2, more rapid post-weaning shifts in LW compared to SW pigs collectively resulted in both dpw- and doa-dependent differences in T-IEL communities in SW versus LW pigs. Significantly different abundance of T-IELs was detected between LW and SW pigs at 3dpw (423 neighborhoods) (Fig. 3D) as well as at 28doa (825 neighborhoods) (Fig. 3E). Results of annotation-independent analysis of T-IEL compositions with an expanded cell surface marker profile were comparable to findings reported in Fig. 2.

### Impact of weaning age on jejunal mucosal microbiota

**Fig. 4:**
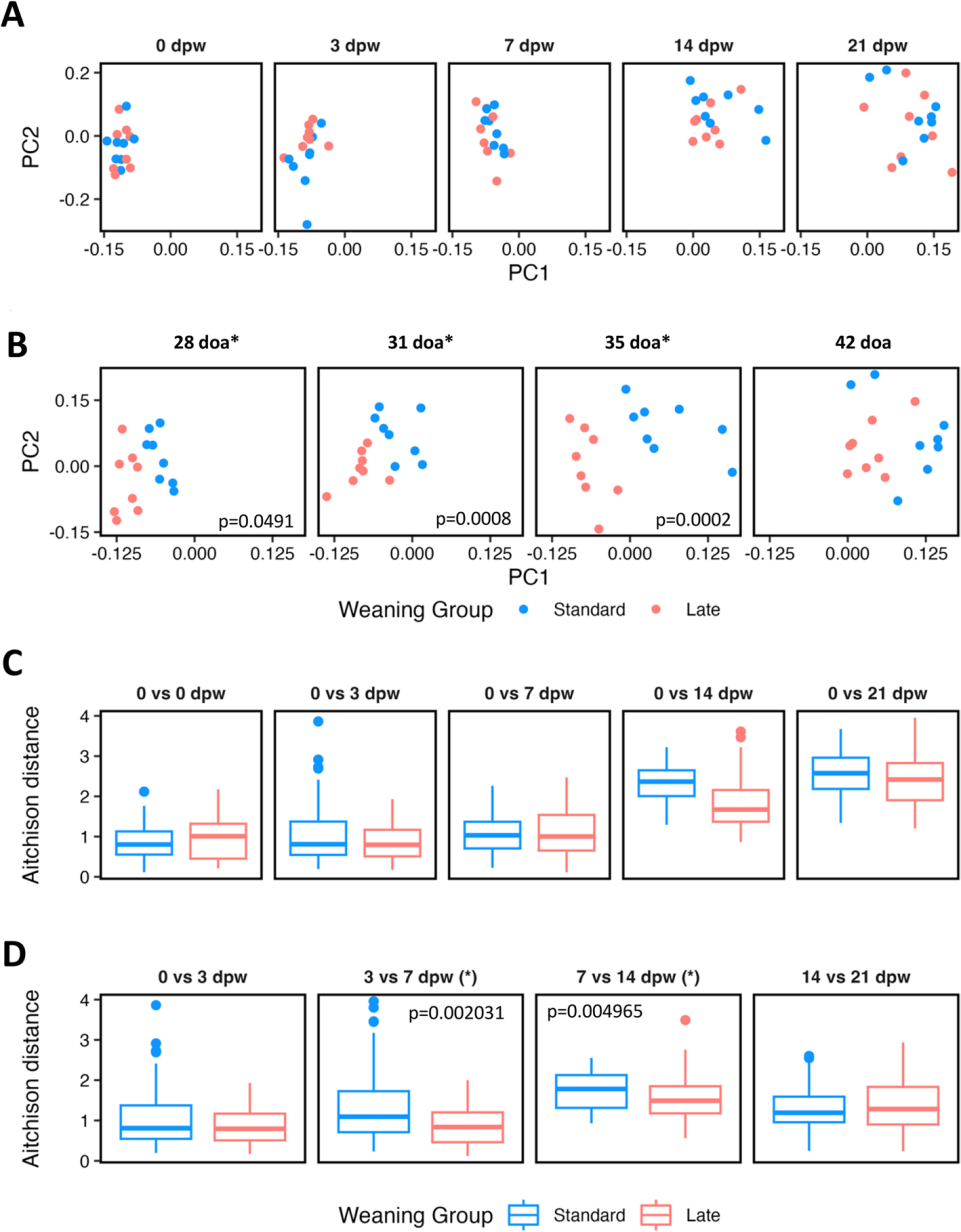
Robust Aitchison PCA ordination and distances to evaluate shifts in mucosal bacterial communities from the jejunum of SW and LW pigs. Ordination of bacterial profiles from the jejunal mucosa of SW (blue) and LW (red) pigs paired on (**A**) dpw or (**B**) doa at necropsy. Asterisks denote statistical significance in pairwise PERMANOVA tests between standard and late-weaned pigs after FDR correction at the comparison time point. **C** Aitchison distances relative to 0 dpw (baseline) were used to visualize shifts in the jejunal mucosa bacterial community composition over time in SW (blue) and LW (red) pigs. Aitchison distances were used to assess the magnitude of the shift in the jejunal mucosa microbiome of SW and LW pigs between subsequent necropsy time points. Asterisks denote statistical significance between SW and LW pigs at the comparison time points. Abbreviations: doa (days of age); dpw (days post-weaning); standard-weaned (SW); late-weaned (LW)

Intestinal T-IELs cells receive important developmental signals from the microbiota and dietary components [reviewed in [7]]. Thus, we investigated changes in the jejunal mucosal bacterial communities through 21dpw in SW and LW pigs (*n* = 8 pigs/weaning age/timepoint, Fig. 4). The mucosal bacterial communities were different based on doa (PERMANOVA, *p* < 0.00001, *R*^2^ = 0.530), but not weaning age group (*p* = 0.305, *R*^2^ = 0.006), sex (*p* = 0.813, *R*^2^ = 0.001), or farrowing pen (*p* = 0.187, *R*^2^ = 0.072) (Fig. 4A-B and Supplementary Fig. 2). Further, there was no significant interaction between the variables encoding weaning age group (LW/SW) and doa. Subsequent pairwise comparisons focused on dpw and doa identified similar findings. The mucosal bacterial communities were not different between SW and LW pigs matched for dpw (pairwise PERMANOVA, FDR corrected *p* > 0.05) (Fig. 4A). However, there were significant differences in the mucosal bacterial communities between SW and LW pigs matched for doa at 28doa (pairwise PERMANOVA, FDR corrected *p* = 0.0491), 31doa (FDR corrected *p* = 0.0008), and 35doa (FDR corrected *p* = 0.0002), but not at 42doa (FDR corrected *p* = 0.1821) (Fig. 4B), suggesting the bacterial communities may have reached a similar composition at this later age timepoint.

Given that age explained most of the variation in the dataset (which is related to the change in diet with weaning), we next examined differences in the longitudinal dynamics of the jejunal mucosal bacterial communities in the SW and LW pigs. Using 0dpw as a baseline to compare against, we noted the largest shift in mucosal bacterial composition first occurred at 14dpw, regardless of the weaning age (Fig. 4C and Supplementary Fig. 2). The SW pigs displayed two significant changes in the jejunal mucosal bacterial communities– from 3 to 7dpw (pairwise PERMANOVA, FDR corrected *p* = 0.0174) and from 7 to 14dpw (FDR corrected *p* = 0.0011). In contrast, the only significant shift in the LW jejunal mucosal bacterial communities occurred from 7 to 14dpw (pairwise PERMANOVA, FDR corrected *p* = 0.0016) (Fig. 4D and Supplementary Fig. 2). We also noted the magnitude of the shifts in the bacterial communities from 3 to 7dpw and from 7 to 14dpw was greater in SW pigs than was observed in LW pigs (Wilcoxon Rank Sum Test, *p* < 0.05) (Fig. 4D).

### Pig weight gain and weaning age

**Fig. 5:**
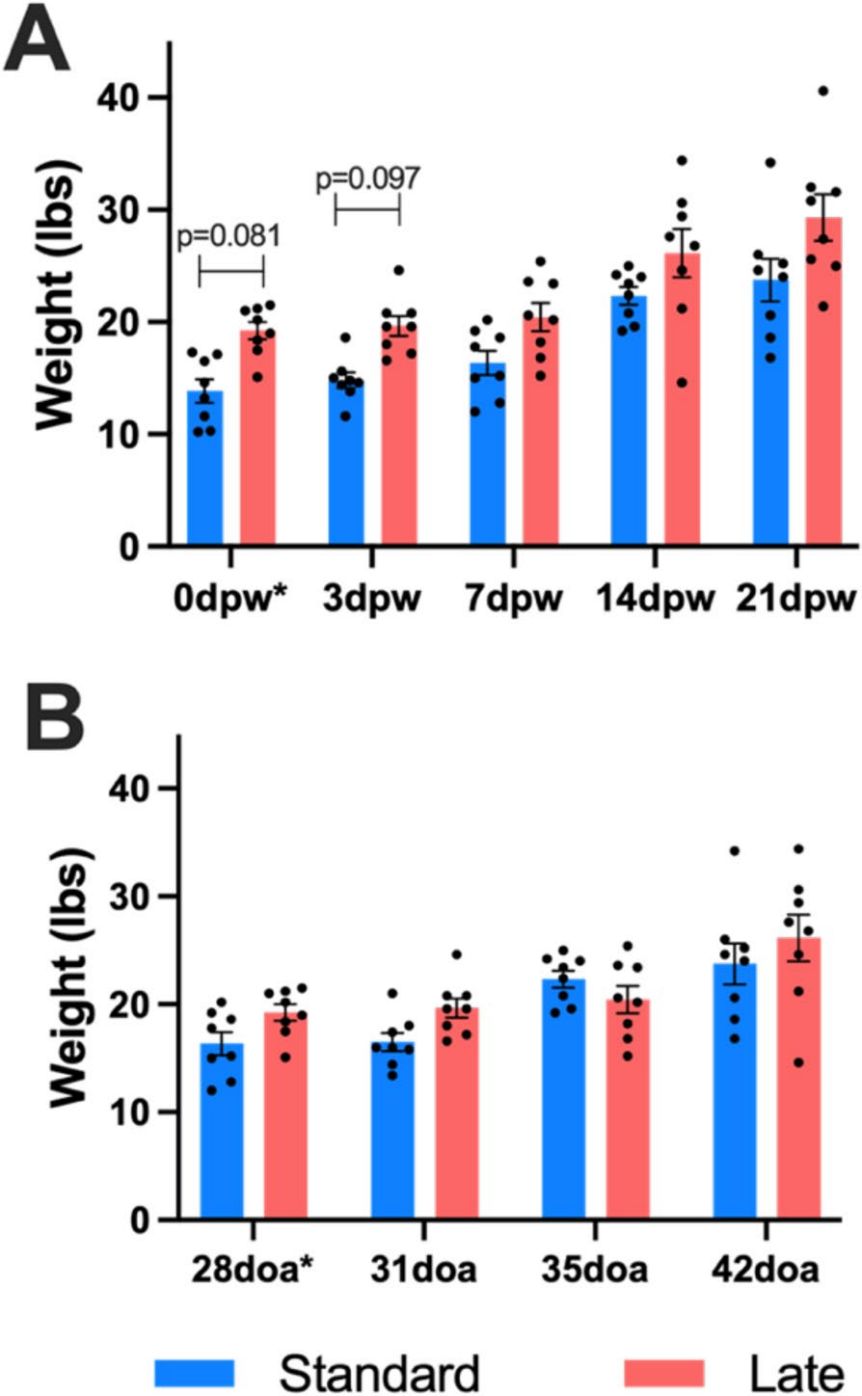
Weight gain is not affected by weaning age. Comparison of weight of pigs in (**A**) dpw-matched timepoints or (**B**) doa-matched timepoints of standard- (blue) versus late- (red) weaned pigs. One day prior to weaning and transport, pigs were weighed for 0dpw (reported as 0dpw*/28doa*). All other dpw, weights were recorded immediately prior to necropsy. Statistical comparisons were performed with a Kruskal–Wallis test then Dunn’s test for selected pairwise comparisons. All *p*-values < 0.10 are shown. Abbreviations: doa (days of age); dpw (days post-weaning)

We established later weaning age affects the rates at which T-IEL and mucosal bacterial communities stabilize in the pig jejunal mucosa and subsequently examined a potential association with pig weights. Pre-weaning pig weights (collected the day before weaning and transport were recorded as 0dpw*) were similar between all timepoints within the SW or LW treatment groups (data not shown). LW pigs weighed more than SW pigs at pre-weaning (0dpw) due to their increased age (Fig. 5A). As age is associated with weight, the LW pigs were heavier than dpw-matched SW pigs, though *p*-values < 0.1 were detected only on 0dpw and 3dpw, and thereafter differences were not significant but trended higher in LW pigs. In regard to doa, weights of SW and LW pigs were similar across doa (Fig. 5B), with line slopes and intercepts not being significantly different (data not shown).

## Discussion

Dietary and environmental changes are two major stresses associated with pig weaning [15, 18] and likely drivers of detected T-IEL and bacterial community shifts in the current study. The structure of the bacterial communities in the jejunal mucosa were the same on the day of weaning (0dpw) between 21doa (SW) and 28doa (LW) groups, indicating age alone prior to weaning did not impact bacterial communities (Fig. 4C). While shifts in the bacterial communities occurred following weaning, they were not directly impacted by age at weaning (Fig. 4A). Differences in the bacterial communities were noted when comparing pigs at the same doa, which captured the impact of time on solid diet between the groups (Fig. 4B). Specifically, at the 28doa comparison, the SW group had been on solid food for 7 days, but the LW group had not yet received any solid food. Early-life feeding has a significant impact on colon microbiota and improves parameters of intestinal health [55]. Thus, offering of creep feed as a pre-weaning nutritional strategy, regardless of weaning age, may modulate and stabilize the intestinal microbiota, potentially minimizing post-weaning intestinal immune and microbial disruptions.

Shifts in jejunal T-IEL populations were associated with time since weaning, regardless of age. Differences in T-IEL populations were not detected for 0dpw but were noted at 3dpw (Figs. 2D-E and 3D) and for the earliest doa timepoint, 28doa (Figs. 2F-G and 3E). Specifically, natural γδ T-IEL populations were highest at weaning, regardless of weaning age, and γδ T-IEL abundances decreased with dpw. A concomitant increase in induced CD8β^+^ T-IELs was noted, which is comparable to prior studies in pigs [10, 11]. While slight differences in jejunal T-IELs between SW and LW groups were noted early after weaning, by 7dpw, both SW and LW groups had stabilized T-IEL populations (Figs. 2B-E and 3C). CD8β + T-IELs are induced in the periphery and increase in the jejunum with exposure to dietary and microbial antigens, which significantly increases with the introduction of solid food and change in environment that occurs with weaning. This likely explains why γδ T-IEL populations were highest at weaning, regardless of weaning age and the dpw was associated with increases in CD8β + T-IELs, as it related to exposure to dietary antigen. Offering creep feed while still suckling is associated with maturation of intestinal immunity [56], although the direct impact of creep feed on T-IEL abundance and function has not been well studied. Moreover, the reported results demonstrated that the majority of T-IEL community shifts occurred in the immediate post-weaning period (within 7 days of weaning), with shifts largely resolving by 3dpw in LW pigs and by 7dpw in SW pigs (Figs. 2B-C and 3C). It is plausible that disruption of jejunal T-IEL populations in SW pigs lasting longer than in LW pigs contributes to a longer post-weaning window of susceptibility to disease or limited intestinal integrity. It’s not uncommon for piglets to eat a limited amount of feed during the first few days in the nursery, another variable that may impact T-IEL abundances and shifts.

Understanding the interactions between the intestinal microbiota and mucosal immune cells is crucial because both play a critical role in maintaining gut homeostasis; however, the interactions are often difficult to dissect. In mice, both dietary and microbial antigens drive the development and abundance of CD8αβ + αβ T-IELs cells, whereas the intestinal microbiota is important for functional maturation of T-IELs [57]. In piglets, addition of solid feed from an early age accelerates both microbiota and intestinal immune maturation, though cell function was not explored [56]. In the current study, the abundances of jejunal T-IELs may not have been dramatically impacted by weaning age due to the dietary change driving the T-IEL population shifts. However, T-IEL function, which was not thoroughly assessed in the current study, could be affected by weaning age [57], as weaning can impact T-IEL activation and metabolism [58, 59]. Collectively, it is difficult to quantify direct and indirect interactions between the microbiota and mucosal immune cells, and methods to better investigate these interactions remain an important area of research [60]. Detailing the concurrent response of the microbiota and mucosal immune populations represents a critical first step towards the development of more mechanistic analyses of early-life interactions between the swine microbiota and immune system, and future studies warrant interrogation of cell function.

The association between gut microbiota and pig performance is a highly investigated area, and methods to enhance the abundance and/or functional activity of specific bacterial populations associated with improved growth performance and health status are desired. Inconsistencies across investigations are noted and likely due to nuanced but important differences across studies [61]. The current study likely lacked sufficient numbers to adequately address changes in performance, and pigs were housed in a research facility as opposed to a more conventional production rearing system. The set up did not allow for assessing feed intake, which may also differ with weaning age. Additionally, the impact of weaning age on performance may not be detectable until pigs face a pathogenic challenge [18, 36, 37]. Thus, our results indicate overall weight gain through the nursery period was not significantly impacted by the weaning ages examined in the current study; however, additional assessment of potential performance benefits may be warranted.

Overall, pig age at weaning did result in changes to the jejunal mucosal bacterial communities and T-IEL populations. As T-IELs play a key role in intestinal homeostasis and barrier integrity, the early differences in population abundance may be indicative of functional differences as well. Bacterial community differences between dpw groups were noted for a longer post-weaning duration (out to 35 dpw) than shifts in T-IEL populations (out to 7dpw), suggesting unique drivers of each variable. While age at weaning impacted T-IEL populations and the structure of bacterial communities, diet and environmental change associated with weaning were more impactful than the seven-day difference of weaning age. Future studies aimed at evaluating T-IEL function as well as abundance will provide deeper insight on the impact of weaning age to pig intestinal health, and challenge studies may be warranted to tease out subtle differences detected under various intervention approaches, including age at weaning.

## Supplementary Information

Supplementary Material 1.

## Data Availability

As noted in Materials and Methods section, scripts used for computational analyses of flow cytometry data are available at https://github.com/SwiVi/FlowCytometry_HighDimAnalysis_TIELs_WeaningAge. Scripts used for computational analyses of 16S rRNA sequencing data are available at https://github.com/USDA-FSEPRU/fs24_16S_jejunum. All raw sequence data has been deposited in the SRA and is available under BioProject PRJNA1199683.

## Abbreviations

ASV: Amplicon sequence variants
Doa: Day(s) of age
Dpw: Day(s) post-weaning
FDR: False discovery rate
LW: Late weaned
SW: Standard weaned
T-IEL: Intraepithelial T lymphocyte
