# Supplementary material for: Weaning age impacts intestinal stabilization of jejunal intraepithelial T lymphocytes and mucosal microbiota in pigs
Source: BMC Vet Res. 2025 Jul 19;21:477. doi: 10.1186/s12917-025-04850-5 (PMC12275383; doi:10.1186/s12917-025-04850-5)
Supplement: Supplementary file 1 — Supplementary Material 1. [file 12917_2025_4850_MOESM1_ESM.docx]

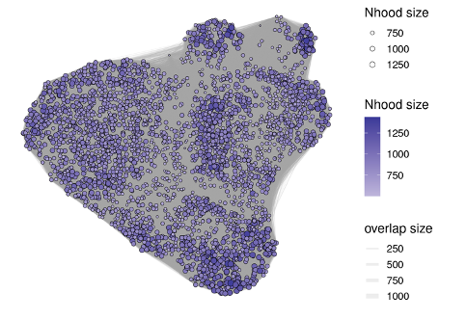


**Supplementary Figure 1. Cell neighborhood groupings used for differential abundance testing of T-IELs.**

Cell neighborhoods used for differential abundance testing overlayed onto UMAP coordinates. Size and fill correspond to the number of cells in a neighborhood. Grey lines connect neighborhoods and have widths corresponding to the number of cells found in both of two connected neighborhoods.

Abbreviations: T-IEL (intraepithelial T lymphocyte); UMAP (uniform manifold approximation projection)

**Supplementary Figure 2.** Robust Aitchison PCA ordination of jejunal mucosa bacterial communities collected at different time points after weaning from SW (a) and LW (b) pigs.
